# Longer-Term Postcure Measurement of Cuspal Deformation Induced by Dimensional Changes in Dental Materials

**DOI:** 10.1155/2015/915071

**Published:** 2015-07-16

**Authors:** A. Falsafi, J. D. Oxman, P.-H. Tse, T. T. Ton

**Affiliations:** 3M ESPE Dental Division, 3M Company, 260-2B-12, 3M Center, Saint Paul, MN 55144, USA

## Abstract

*Aim*. This paper presents a simple, versatile in vitro methodology that enables indirect quantification of shrinkage and expansion stresses under clinically relevant conditions without the need for a dedicated instrument. *Methods*. For shrinkage effects, resulting cusp deformation of aluminum blocks with MOD type cavity, filled with novel filling compositions and commercial cements, has been measured using a bench-top micrometer and a Linear Variable Differential Transformer (LVDT, a displacement transducer) based instrument. *Results*. The results demonstrated the validity of the proposed simple methodology. The technique was successfully used in longer-term measurements of shrinkage and expansion stress for several dental compositions. *Conclusions*. In contrast to in situ techniques where a measuring instrument is dedicated to the sample and its data collection, the proposed simple methodology allows for transfer of the samples to the environment of choice for storage and conditioning. The presented technique can be reliably used to quantify stress development of curing materials under clinically relevant (oral) conditions. This enables direct examination and comparison of structural properties corresponding to the final stage of formed networks. The proposed methodology is directly applicable to the study of self-curing systems as they require mouth-type conditions (temperature and humidity) to achieve their designed kinetics and reactions.

## 1. Introduction

Shrinkage stress of dental material has attracted the attention of many clinicians and researchers. However, most of the research in this field has been confined to testing and methods that have addressed measurements during short intervals under conditions different from the oral environment. It has been well documented that during curing and setting reactions of restorative materials there are sizable dimensional changes that lead to high level of interfacial stresses at the confining hard boundaries [1, 2]. The decrease in overall system's entropy, associated with the ordering rendered by formed polymeric network(s), has been the driving force for such stresses. Adhesive dentistry has been developed to combat such forces and ensure intimate contact at such interfaces to provide acceptable marginal integrity. Additionally, incremental or layering technique has been employed to mitigate shrinkage related forces. In recent years, there has been significant activity in the development of low stress compositions to enable bulk-filling/curing as a faster and easier technique. Several new entries to the market, based on different monomer systems and/or reaction schemes, claim to have created low shrinkage stress. Recently, it has been shown that the inclusion of relatively small amount of Addition-Fragmentation Monomers (AFM) can significantly reduce the polymerization stress in purely acrylate networks. The inclusion of this class of monomers into the polymerization mixture enables the network to rearrange, that is, adapt during and/or after the polymerization, to accommodate the shrinkage without developing significant stress [3, 4].

It is known that network formation is a lengthy process that can take hours. That is why most, if not all, property testing protocols call for 24 hours' storage under oral conditions. Although there has been extensive report on mechanical and interfacial properties of dental materials and their evolution with time [1, 5], there have been very few, if any, publications for longer-term development of shrinkage stress in a quantitative manner. Unfortunately, the data in the literature related to this subject have been mainly limited to a time frame of minutes, either due to instrument limitation with respect to environmental compatibility or due to the measurement drift associated with the electronic components in the long-term use [1, 6–10].

While for filling materials high level of shrinkage stress has been shown to impact long-term performance and longevity of direct restorations, compressive stresses due to excessive hygroscopic expansion of cements have been implicated in failure and fracture of crowns and indirect articles. The network formed from hydrophilic monomer systems has greater potential for water uptake and expansion upon exposure to oral environment. Similar to shrinkage phenomenon, hygroscopic expansion can take days to mature and the resulting stresses, if not compensated by simultaneous shrinkage effects, can be sizable and detrimental to the surrounding structures. Again, there has been no report of quantitative measurements of expansion stress for dental materials and its longer-term evolution with time under clinically relevant conditions.

Aluminum blocks with MOD (Mesial-Occlusal-Distal) cavity have been successfully used to quantify shrinkage effects associated with dental filling compositions. Aluminum has a modulus of elasticity (ca. 70 GPa) close to that of enamel and represents a consistent material to reduce inherent variations in natural tooth mechanical properties [11]. The cuspal deformation (strain) at the edge of the thin walls of aluminum block (measured in microns) is equal to the cuspal stress (measured in MPa) when divided by aluminum's modulus of elasticity. The aluminum cuspal stress is equal to the shrinkage stress of the filling material transmitted across the wall interfaces.

The purpose of this study is to establish a simple, fast, and cost-effective methodology that enables measurement of cuspal deformations mediated by the stresses associated with dimensional changes of dental compositions, during and after polymerization, under oral conditions.

## 2. Materials and Methods

### 2.1. Compositions

**Figure 1:**
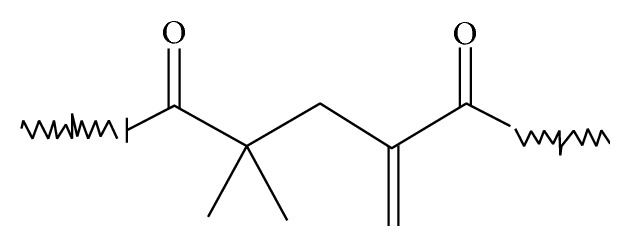
Generic depiction of Addition-Fragmentation Monomer (AFM) used in the experimental filling composition.

Table 1 shows the two-part dental filling and cement compositions that were used for this study. The dual-cure experimental filling compositions contained different levels of stress-relieving Addition-Fragmentation Monomer (AFM). The generic structure of the used AFM has been depicted in Figure 1. It is believed that the fragmentation of the addition-fragmentation crosslinking agent (between the tertiary carbon and the unsaturated C=C) provides a mechanism for crosslink cleavage. The cleavage of labile crosslinks may allow the polymeric network to relax or reorganize, especially in high stress regions, providing a potential mechanism for stress relief [12]. The cement compositions were commercial luting cements with different chemistries.

### 2.2. Aluminum Block and Its Surface Treatments

**Figure 2:**
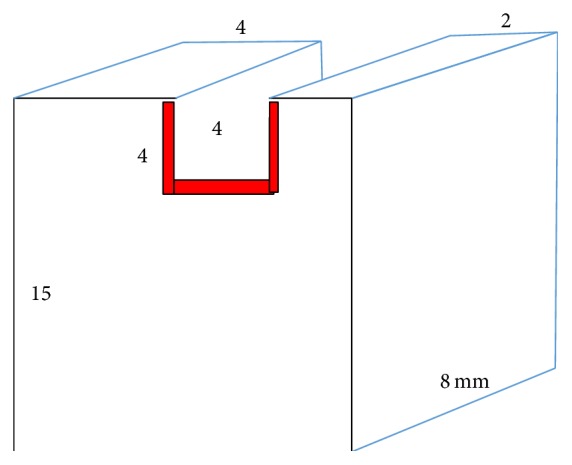
Geometry of cusp deformation block and its cavity. Standard cavity depth of 4 mm has been presented.

Aluminum blocks (Extrusion Alloy, Al > 98%) were machined per geometry illustrated in Figure 2. For hygroscopic expansion measurements, the blocks were anodized (in blue to be nonabsorbing to blue LED light) to prevent/minimize corrosion while immersed in water. The following surface treatments were performed to ensure maximum interfacial compatibility with the loaded methacrylate based compositions. The inner surface of the cavity was sandblasted (Sand Storm by Vaniman Manufacturing Co.) to increase the surface area for bonding. A thin layer of ceramic primer solution (3M ESPE RelyX Ceramic Primer) was brushed onto this inner surface and dried prior to application of the adhesive (3M ESPE Adper Easy Bond Self-Etch Adhesive). The adhesive was applied with a brush and then gently air-dried and cured with a halogen light, all per instructions for use. The adhesive is a methacrylate based composition that ensures full compatibility and adherence to the tested (methacrylate based) compositions in the shrinkage studies.

In order to contain the starting flowable compositions within the cavity, Scotch Tape was applied on end surfaces. In one study of filling compositions, the cavity depth and block height were increased by 2 mm beyond what is shown in Figure 2. For hygroscopic expansion study of cements, 2 mm wide and 2.5 mm deep cavity blocks were used. Three blocks were used for each sample.

### 2.3. Cusp Deformation Measurement Using LVDT-Based Instrument

In this technique, LVDT (a displacement transducer) RDP 93062 along with RDP Transducer Indicator E309 (RDP Electrosense, Pottstown, PA) with a repeatability of less than 0.2 microns was used to measure the cusp deformation (deflection of the thin wall of the cavity). Upon loading the composition in the block cavity and subsequent clamping of the block in place, the transducer's stylus was brought into contact with the edge of the cusp. After an equilibration period of about 5–10 minutes per sample, a baseline was established for 1 minute and cusp deformation measurements were collected as the composition was self-cured and/or light-cured. In light-curing mode, the blue LED curing light (~1200 mW/cm^2^) was turned on for 1 minute and cusp deformation was continuously measured for an additional duration of 10 minutes using the LVDT strain gauge. These measurements were limited to the constant room conditions of 24°C and 50% RH. The description of the design has been previously disclosed [11].

### 2.4. Cusp Deformation Measurement Using Micrometer

**Figure 3:**
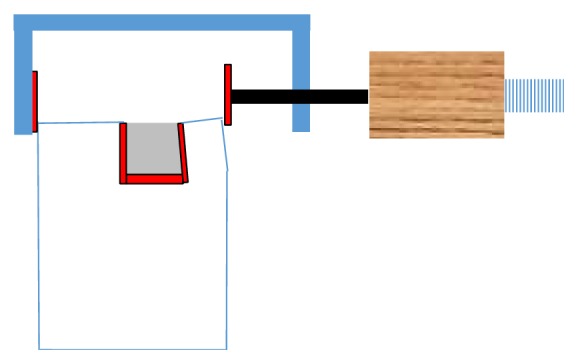
Illustration of shrinkage-induced cusp deformation and the measurement tool. Micrometer contact surface and line are shown.

Mitutoyo Coolant Proof Micrometer Series 293 (resolution of 1 micron, manufacturer number 293-330) was used for the two-ended measurements according to the following protocol. The micrometer jaws were gently brought into contact with the edges of cusps in a manner where the area of contact was very small and limited to the outer edges as shown in Figure 3. Only 2 clicks of the fine screw were used to establish the final contact. Three such measurements were made for each block and the average was used as reference (*t* = 0).

After injecting compositions in the cavity, the above measurements were repeated to ensure repeatability and ruling out any block contamination during composition loading in the measurement areas. The loaded blocks were transferred to an oven at 37°C and 95% relative humidity to allow for continuation of cure and equilibration mimicking the oral environment. At desired time intervals after initial measurements, the samples were taken out of oven and brought to room temperature (24°C and 50% RH) for 15 minutes before cusp deformation measurements. Afterwards, the samples were returned to the oven until next scheduled measurement. In case of luting cements, they were conditioned for 1 hr in 37°C and 95% relative humidity chamber and then transferred to 37°C deionized water for extended storage.

## 3. Results/Discussion

### 3.1. Methodology Validation

**Figure 4:**
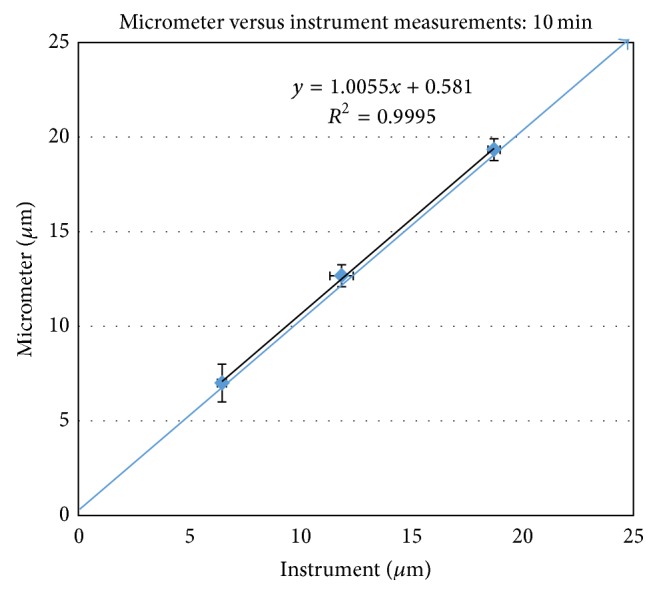
Cuspal deformation of Al blocks measured by bench-top micrometer versus LVDT instrument as a result of polymerization stress.

Figure 4 demonstrates the equivalency of “micrometer-based” and “instrument-based” methods in measuring cusp deformation due to shrinkage stress. The samples were light-cured per protocol described in the Methods. Results demonstrate excellent agreement between the two methods studied. It should be noted that since all the shrinkage/expansion measurements were done under controlled (24°C and 50% RH) conditions, no net thermal effects were expected. In the case of shrinkage stress measurement for light-curing compositions, the thermal effects due to exothermic polymerization were seen to dissipate very quickly after the light-curing unit was turned off (few minutes). Therefore, the final readings (10 minutes) were not impacted by such transient thermal expansion effects of the system.

### 3.2. Examples of Cuspal Deformation Measurements Induced by Shrinkage Stress

**Figure 5:**
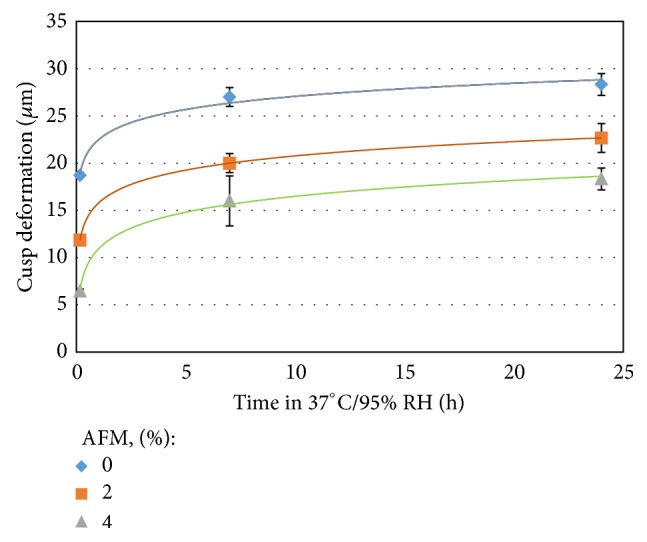
Change of cuspal deformation of Al blocks measured by micrometer as a function of postcure time and stress-relieving component (AFM) content in the final mix.

Figure 5 shows the application of the technique in quantifying the short- and long-term change in cuspal deformation after light-curing. The experimental two-part dual-curing composition comprised various levels (w/w) of stress-relieving component, namely, Addition-Fragmentation Monomer (AFM). The plot of cuspal deformation versus time over 24 hrs shows a logarithmic pattern. As it can be seen, upon reaction initiation, there is a rapid change in stress level. However, the long-term measurements show a cuspal deformation plateau after a few hours, suggesting attainment of full possible conversion for the given compositions within this period of time [1]. Both the initial and long-term stresses were impacted by the presence of stress-relieving component known as Addition-Fragmentation Monomer (AFM) in the formulation suggesting a sustained relief of stress.

**Figure 6:**
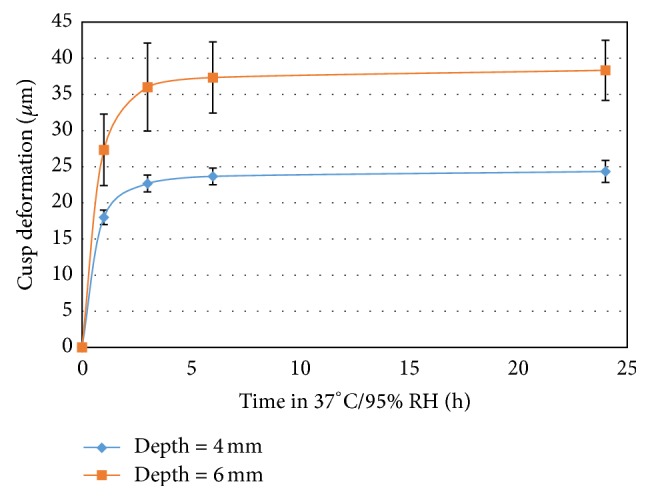
Cuspal deformation as a function of time and cavity depth.

Figure 6 confirms the increase in shrinkage stress induced cuspal deformation for deeper cavity when the dual-curing composition above (AFM = 2%) was solely self-cured. The use of a self-cure system affords a more uniform profile of conversion, within the cavity, in contrast to a light-curing one where the results would have been confounded due to spatial (depth) variations in conversion corresponding to light penetration and absorption. Further light-curing of the samples resulted in cusp deformations less than 1 micron indicating that final potential conversion was achieved in self-cure mode for these samples after 24 hrs at 37°C. The proportional increase in cuspal deformation with depth suggests a bulk property dependency which may not be the case for purely light-curing systems (e.g., one-part compositions) where polymerization profile is biased towards areas close to the light.

### 3.3. Examples of Expansion Measurements

In another experiment, the technique was used to quantify cuspal deformations related to hygroscopic expansion of different luting dental cements. This group of blocks was immersed in 37°C DI water for the duration of the study. In order to prevent corrosion of blocks, they were anodized prior to experiment. An empty aluminum block group was added as a negative control.

**Figure 7:**
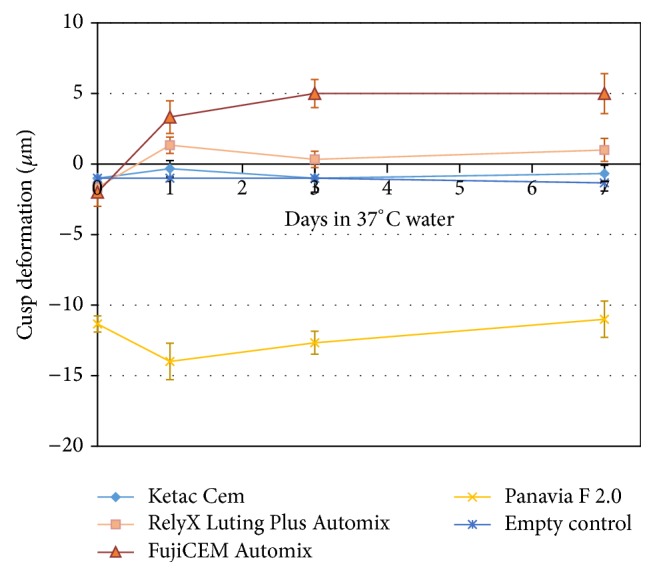
Cuspal deformation during curing and after days of immersion in 37°C water: short-term shrinkage versus longer-term expansion of cements.

Luting cements constituents differ significantly in their chemistries and corresponding cured networks which impacts their hydrophilicity and water-uptake properties. While resin cements (e.g., Panavia F 2.0 by Kuraray Dental) comprise more hydrophobic monomers, conventional and resin-modified glass-ionomer cements are water-based and quite hydrophilic. Not all glass-ionomer cements have the capability to absorb significant amount of water as this is also impacted by the final cement network and its swelling potential. In contrast to conventional glass ionomer (e.g., Ketac Cem by 3M ESPE) which has solely an inorganic (polysalt) network, resin-modified glass ionomers (RMGI, e.g., RelyX Luting Plus by 3M ESPE and FujiCEM by GC) additionally have a hydrophilic resin network which gives them a swelling potential when exposed to water. The data captured in Figure 7 suggest an initial stage of curing dominated by shrinkage stress (within few hours for RMGI cements, versus 1 day for the resin cement) followed by a hygroscopic expansion matured within days. Also as expected, the hydrophobic resin cement was dominated by shrinkage effects at the early stage of curing, and there were no statistical changes for the empty block and the conventional glass-ionomer cement.

In a clinical application, cements are used as thin layers (generally less than 100 microns) to lute or bond articles such as crowns or inlay/onlays to the (tooth) abutments. Although in our study cements were filled in the cavities, this does not change the comparative conclusions made above.

**Figure 8:**
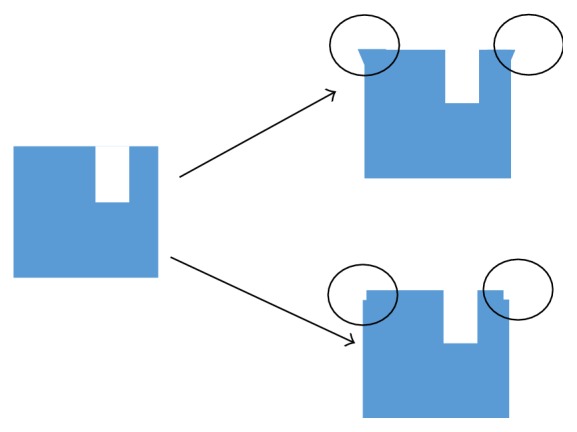
Examples of block-edge design modifications to improve measurement accuracy and repeatability.

The proposed method, if needed, can be further improved to provide more accuracy and repeatability via the use of commercially available higher precision (submicron) micrometers and/or blocks with modified edges to ensure line contacts with micrometer contacting plates as illustrated in Figure 8.

## 4. Conclusions

The presented methodology can be reliably used to address stress development of curing materials under clinically relevant conditions. This simple and versatile test enables direct examination and comparison of structural properties corresponding to the final stage of formed networks. To the best of our knowledge, this is the first study to address longer-term postcure shrinkage/expansion stress for dental materials when curing is done per clinically relevant conditions/configuration. The proposed methodology is directly applicable to the study of self-curing systems as they require mouth-type conditions (temperature and humidity) to achieve their designed kinetics and reactions.

## Figures and Tables

**Table 1:** Description of the experimental and commercial materials based on manufacturer's safety data sheets.

Material	Manufacturer	Chemistry	Batch number
RelyX Luting Plus Automix	3M ESPE	Paste/paste resin-modified glass-ionomer (RMGI) cement Methacrylated polyacid, water, fluoroaluminosilicate (FAS) glass, methacrylated monomers, and initiators	L: N305342 EXP: 2013-07

GC FujiCEM Automix	GC	Paste/paste RMGI cement Polyacid, water, FAS glass, methacrylated monomers, and initiators	L: 1003191 EXP: 2012-03

Ketac Cem Aplicap	3M ESPE	Powder/liquid conventional glass-ionomer cement Polyacid, water, and FAS glass	L: 400845 EXP: 2013-08

Panavia F 2.0	Kuraray	Paste/paste dual-curing resin-based adhesive cement 10-Methacryloyloxydecyl dihydrogen phosphate (MDP), hydrophobic methacrylated monomers, silanated silica and barium glass fillers, and initiators	L: 61112 EXP: 2013-01

Adper Easy Bond	3M ESPE	Self-Etch Adhesive Water, ethanol, methacrylated polyacid, methacrylated monomers, phosphoric acid-6-methacryloyloxy-hexylesters mixture (MHP), silane treated silica, and initiators	L: 418381 EXP: 2012-10

RelyX Ceramic Primer	3M ESPE	Ceramic primer Water, ethanol, and methacryloyloxypropyl-trimethoxy silane	L: N105093 EXP: 2012-08

Experimental compositions	3M ESPE	Paste/paste dual-curing composite filling Methacrylated monomers, varying Addition-Fragmentation Monomer (AFM) levels, fillers, and initiators	NA
